# Improving the compliance of orthopaedic wrist and hand referrals against the musculoskeletal recommendations from the 2018 Evidence-based Interventions programme, along with local guidance in Greater Manchester: A quality improvement project

**DOI:** 10.1136/bmjoq-2025-003323

**Published:** 2025-09-26

**Authors:** Dylan L Woodhead, Peter C Goodwin, Eula Miller

**Affiliations:** 1 Health Professions, Manchester Metropolitan University, Manchester, UK; 2 Cora Health, Newcastle upon Tyne, Tyne and Wear, UK; 3 Department of Health Professions, Manchester Metropolitan University, Manchester, England, UK; 4 School of Nursing and Public Health, Manchester Metropolitan University, Manchester, England, UK

**Keywords:** Waiting Lists, Surgery, Quality improvement, PDSA, Patient-centred care

## Abstract

**Background:**

The National Health Service Long-Term Workforce Plan calls for improving clinical pathways for surgery. Four wrist and hand surgeries, including carpal tunnel syndrome release, Dupuytren’s contracture release, ganglion excision and trigger finger release, are described as procedures of limited clinical value and are included in the 2018 Evidence-based Interventions programme, as well as local guidance in Greater Manchester (GM).

**Local problem:**

A pre-scoping exercise audit at a single musculoskeletal service in GM conducted from May 2021 to June 2023 highlighted that clinician compliance rates for these referrals were 15% below the service provider’s internal national average and 25% below the service provider’s internal national target, demonstrating the need for a quality improvement project.

**Methods:**

The Model for Improvement was implemented using four Plan–Do–Study–Act (PDSA) cycles. These cycles were executed over 14 weeks and aimed to improve compliance through educational sessions, clinical resources, interactive learning and practical tools.

**Intervention:**

The project comprised four PDSA cycles: PDSA 1 introduced educational sessions and case discussions, PDSA 2 implemented a clinical flowchart to guide decision-making, PDSA 3 included a knowledge retention quiz and PDSA 4 involved a repeat quiz and further discussions to consolidate learning. The target was to increase compliance rates from 70% to 85% or more.

**Results:**

The project successfully improved compliance rates by 30%, with the final compliance rate reaching 100%, surpassing the service provider’s internal national average and target, respectively. 100% compliance was achieved and sustained during PDSA 4 until the end of the project. Clinician confidence and quiz scores also increased during the intervention.

**Conclusions:**

Educational initiatives, combined with practical tools like clinical flowcharts and quizzes, significantly improved compliance rates. The project provides a scalable model that can be adapted by other community healthcare services to enhance compliance with orthopaedic referrals.

**WHAT IS ALREADY KNOWN ON THIS TOPIC**

- There is limited evidence on the compliance of orthopaedic referrals from community care, specifically for wrist and hand conditions, and this may be due to research focusing primarily on secondary care. There are no randomised controlled trials for this topic and no quality improvement projects exploring the compliance of wrist and hand referrals. The audit highlighted that the clinician compliance rate for referrals to orthopaedics for the four wrist and hand conditions was 70%, which was 15% and 25% below the service provider’s internal national average and internal target set for these conditions, respectively.

**WHAT THIS STUDY ADDS**

- The project provides a model that can be adapted by community musculoskeletal services to improve orthopaedic referral compliance.

**HOW THIS STUDY MIGHT AFFECT RESEARCH, PRACTICE OR POLICY**

- Educational initiatives, combined with practical tools like clinical flowcharts and quizzes, significantly improved compliance rates. The findings were presented ahead of the annual Greater Manchester policy steering group meeting for the four wrist and hand conditions.

## Introduction and background

The single musculoskeletal (MSK) service provides care across the Greater Manchester (GM) region and is part of a larger network of MSK services run by a service provider. In 2023, the single MSK service audited its referral processes, specifically focusing on wrist and hand surgeries classified as procedures of limited clinical value (PLCV).^1^ The four wrist and hand surgeries in question, including carpal tunnel syndrome (CTS) release, Dupuytren’s contracture release, ganglion excision and trigger finger release, fall under the national 2018 Evidence-based Interventions (EBI) programme.^2^ The programme was developed to standardise care, reduce clinical variability and ensure that appropriate cases are referred for surgery. The programme is supported by the National Institute for Health and Care Excellence (NICE).^3^

The EBI programme aims to reduce unnecessary surgeries while improving overall patient outcomes. GM’s local guidance aligns with the EBI programme. Despite the availability of both national and local guidelines, the prescoping exercise audit conducted between May 2021 and June 2023 revealed that referral compliance for wrist and hand conditions at the single MSK service was significantly lower than the service provider’s internal national average. The internal national average was calculated from 11 other single MSK services across the UK within the same provider (n=128). The audit found that compliance rates for these referrals stood at just 70% within the single MSK service, falling 25% short of the service provider’s internal national target of 95%. This discrepancy highlighted the need for an intervention to improve compliance with referral standards.

### Problem description

The audit highlighted several challenges within the single MSK service, most notably a failure to consistently adhere to the referral guidelines outlined in the EBI programme and local guidance. This inconsistency resulted in inappropriate referrals, leading to delays in patient care and additional pressure on secondary care services. The failure to fully explore conservative management options before referring patients for surgery was a key issue, particularly in cases where non-surgical treatments like splinting or corticosteroid injections (CSIs) could have been considered. Incomplete documentation and inadequate adherence to clinical pathways contributed to inefficiencies, increasing the likelihood of patients being placed on surgical pathways without proper consideration of all treatment options.

### Available knowledge

Research specific to improving the compliance of wrist and hand orthopaedic referrals is limited, with much of the existing literature focusing on general orthopaedic referrals or secondary care pathways. Burn and Beeson^4^ reported an 80.5% compliance of an unspecified guidance for orthopaedic referrals in the UK; however, the study did not isolate wrist and hand conditions for analysis. Similarly, studies examining referral processes largely focus on secondary care, and not referral from primary or community care settings.

NICE’s Clinical Knowledge Summaries provide direction for individual conditions such as CTS and Dupuytren’s contracture. The lack of awareness of EBI guidance for ganglions and trigger fingers has likely contributed to variability in clinical decision-making across different healthcare providers. This further emphasises the need for localised quality improvement projects (QIPs) like this, which are designed to address these gaps by standardising referral processes in community MSK services.

Wildin *et al*^5^ found a 36% increase in hand surgery referrals in the UK over a 10-year period from 1990 to 2000; however, these findings might not be transferable as they are outdated and only specify hand conditions. In addition, Dean *et al*^6^ found that non-traumatic wrist and hand conditions represent a significant proportion of new patient referrals, follow-up and treatment in secondary care. This UK study captured data from 160 patients across 16 participating hospitals nationwide; however, they had a wider scope of diagnoses that included 31% osteoarthritis cases, which was not a diagnosis included in the QIP.

### Rationale

The National Health Service (NHS) Long-Term Workforce Plan^7^ highlights the importance of optimising clinical pathways to improve patient outcomes, reduce unnecessary referrals and improve efficiency. In GM, wait times are typically an average of 20 weeks, which is 2 weeks beyond the national target of 18 weeks.^8^ Reducing unnecessary referrals for surgeries classified as PLCV, such as the four wrist and hand surgeries, could significantly decrease these wait times by ensuring that only clinically appropriate cases are referred to secondary care. Moreover, high recurrence rates for certain surgeries such as CTS, where up to 12% of cases require repeat surgery,^9^ which further strain healthcare resources. Furthermore, the northwest of England has a 2.2% higher than average percentage of chronic MSK problems,^10^ which adds further burden to the health system in the north west. These figures are an important part of the rationale and emphasise the need to improve the compliance of referrals to orthopaedics for these conditions by implementing guidance and person-centred care.^11^ Ensuring that conservative treatments are fully explored before surgical referral can mitigate this issue. By improving the compliance of referrals, the QIP aimed to streamline patient pathways, reduce unnecessary surgeries and optimise resource use across GM.

### Specific aims

The primary aim of the QIP was to increase clinician compliance with national and local referral guidelines for wrist and hand conditions from 70% to at least 85% by June 2024, starting in March 2024 and running for a total of 14 weeks. A secondary aim was to improve clinician confidence in applying these guidelines, ensuring the sustainability of the improvements made through the project. Additionally, the project aimed to create a replicable model for improving referral compliance that could be adopted by other community MSK services across the NHS.

## Methods

### Context

The single MSK service is a multidisciplinary service consisting of advanced practitioners, sport and exercise medicine (SEM) consultants and SEM registrars. The team is responsible for assessing patients with MSK conditions and determining whether they should be referred for orthopaedic surgery. Generally, clinical guidance is followed on a case-by-case basis using both local pathways and NICE guidance,^12^ and although local GM policies are used for other MSK conditions, policies for the four wrist and hand conditions are less used. The audit revealed that clinicians were not consistently following the national and local guidelines for wrist and hand conditions, leading to incomplete or inappropriate referrals.

To address these challenges, the project team used the Model for Improvement (MFI) to structure the quality improvement initiative. The MFI tool includes a series of Plan–Do-Study-Act (PDSA) cycles^13^ that enable testing of changes, allowing the team to assess the effectiveness of each intervention before proceeding to the next phase. A Gantt chart was used to outline the project timeline and key milestones, ensuring that each phase was carefully planned and executed. A fishbone (Ishikawa) diagram^14^ was employed to identify the root causes of non-compliance, including clinician unfamiliarity with guidelines, lack of access to clinical resources and time constraints during assessments. All 12 clinicians were provided with a questionnaire to complete.

### Interventions

Following the audit, the MFI tool was used which included four PDSA cycles, running for a total of 14 weeks. There were no data between cycles as each cycle transitioned into the next. The interventions were designed to address the identified gaps in compliance within the single MSK service team through education, practical tools and interactive learning opportunities. Each cycle focused on a specific aspect of improving the referral process.

#### PDSA 1

The first cycle was 3 weeks in duration and introduced an educational session that reviewed the audit findings and introduced both the EBI programme and the GM local guidelines. Clinicians participated in case discussions, which helped clarify key criteria for referral, such as the appropriate use of nerve conduction studies in diagnosing CTS and the clinical thresholds for referring patients with Dupuytren’s contracture for surgery. There was also an opportunity to discuss the benefits of shared decision-making (SDM),^15^ and the orthopaedic NHS decision support tools^16^ available for CTS and Dupuytren’s.

#### PDSA 2

**Figure 1:**
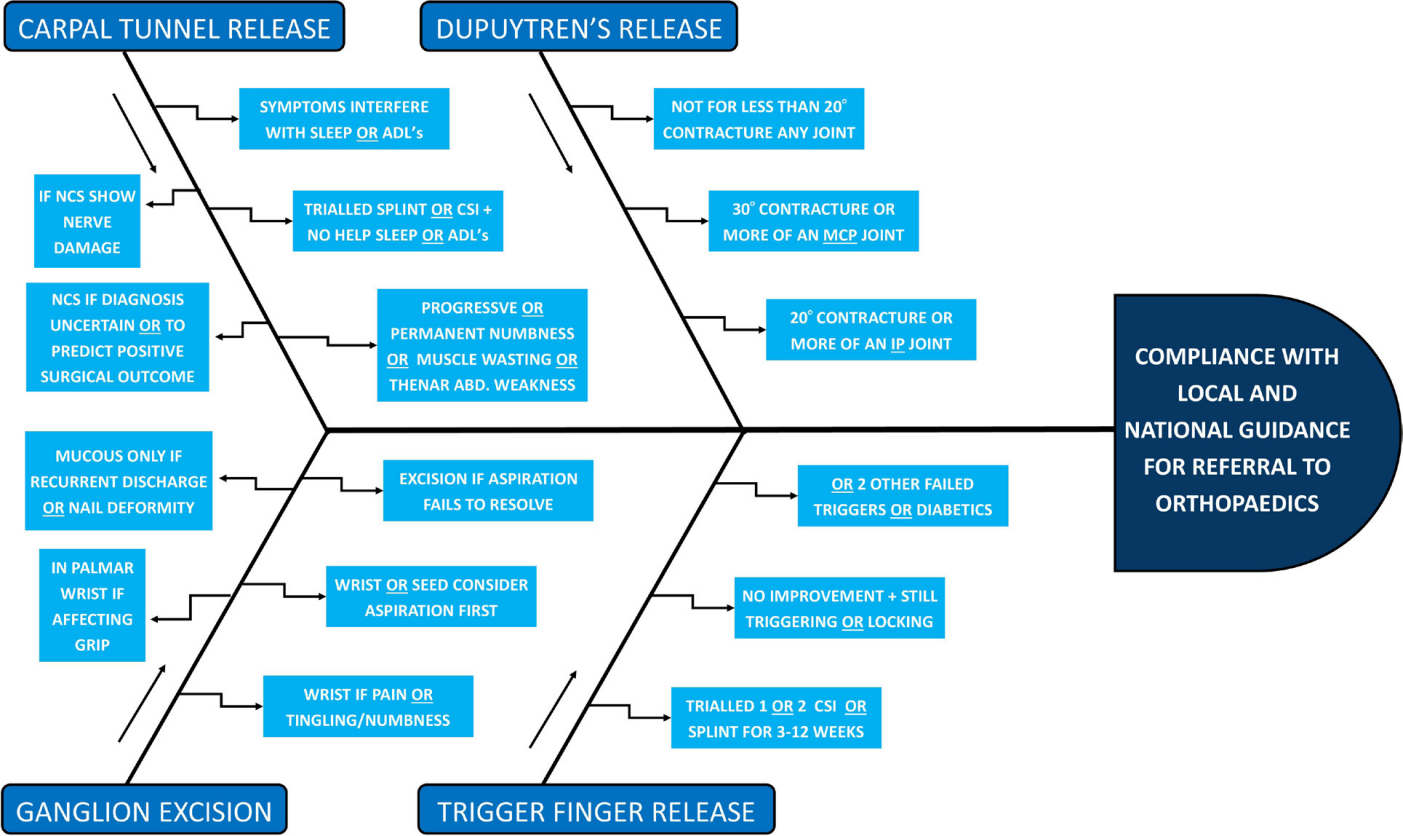
Clinical flowchart for referral to orthopaedics. CSI, corticosteroid injections; NCS, nerve conduction studies; ADL, activities of daily living; ABD, abduction; MCP, metacarpal; IP, interphalangeal.

The second cycle was 3 weeks in duration. Based on feedback from the first session, the team developed a digital folder containing key resources, including a clinical flowchart that outlined the referral criteria for the four wrist and hand conditions. The flowchart (see figure 1) was designed to simplify the decision-making process by providing a quick-reference tool that clinicians could use during consultations. The digital folder was made accessible to all clinicians, ensuring that they had the resources they needed to make appropriate referrals.

#### PDSA 3

The third cycle was 3 weeks in duration and introduced a quiz to test clinician knowledge of the guidelines and their ability to apply them to clinical cases. The quiz (see online supplemental material) was an alternative delivery method which was found to enhance learning.^17^ The quiz focused on the four conditions and enabled discussions of clinical scenarios to challenge clinician understanding of the referral criteria. Additional case discussions were held following a review of the quiz results and addressed any areas of uncertainty.

#### PDSA 4

The final cycle was 5 weeks in duration and involved a repeat of the quiz to assess improvements in knowledge retention and confidence. Additional case discussions were held to reinforce key points from the guidelines and ensure that clinicians felt confident in applying the referral criteria. A Q&A session was included to address any remaining gaps in understanding. There was positive feedback for documenting a diagnosis of diabetes in an orthopaedic referral for trigger finger. This is advised in the EBI guidance and was supported by a meta-analysis by Chang *et al*^18^ which found higher recurrence rates in diabetics following CSI for trigger finger.

### Study of the intervention

The interventions were designed to engage clinicians and encourage active participation. There is limited evidence for change interventions on this topic; however, a study by Curtin and Yao^19^ looked at how hand surgeons could improve their referrer’s understanding of hand surgery through teaching sessions. These education sessions were traditional lecture-based teaching sessions, which tend to encourage more passive learning.^20^ For the QIP, small group teaching was chosen as the primary method of education as it allows for interactive learning and real-time feedback.^21^ The use of quizzes provided a measurable way to track improvements in knowledge retention and application. Case discussions offered clinicians the opportunity to ask questions, discuss complex cases and receive immediate feedback from their peers.

The project team used a range of quality improvement tools, including the Gantt chart, fishbone diagram and driver diagrams, to ensure that the project was well-organised and focused on addressing the key drivers of non-compliance. Control charts were used to track compliance rates over time and identify any shifts in performance.

### Measures

All measures aimed to be specific to the project, measurable with compliance rate data, achievable in the 14-week timeframe, realistic with the data and resources available and timely by setting targets for completion.^22^ The measures were used to assess the success of the project.

#### Outcome measure (measuring improvements)

The primary outcome measure was the compliance rate for wrist and hand referrals to orthopaedics. The target was to increase compliance from 70% to at least 85% from March to June 2024. Achieving this target would demonstrate the success of the project.

#### Process measures (measuring the process)

The first process measure included clinician confidence levels, which was measured using pre-QIP and post-QIP questionnaires. The second process measure involving quiz scores was used to assess improvements in knowledge retention and application.

#### Balance measure (measuring other dimensions)

The first balance measure tracked the percentage of referrals to orthopaedics for the four wrist and hand conditions. The second balance measure tracked referrals to injection clinics for conditions like CTS and trigger finger as injection therapy is a viable alternative to surgery for some cases. An increase in injection clinic referrals would suggest that clinicians were becoming more confident in managing cases conservatively, reducing the need for surgical referrals, and therefore contributing to the overall efficiency and cost-effectiveness in clinical pathways.

Data were analysed using Statistical Process Control (SPC) charts for completeness and accuracy of the data.

### Analysis

Data were analysed using SPC charts due to the amount of data (n=58), which tracked compliance rates over the 14-week period. The Nelson rules^23^ were applied to identify any special cause variations in the data, indicating whether the changes observed were statistically significant. Control charts were also used to track clinician confidence levels and quiz scores, helping identify trends and measure the effectiveness of the interventions.

Quantitative data for the outcome measure were analysed to draw on inferences for clinical compliance. Qualitative data from the process measures were analysed for comparison and to draw on inferences on clinician confidence and knowledge retention. Changes were observed during each PDSA cycle; therefore, the effects of time were analysed throughout the 14-week process. The Health Research Authority tool^24^ was used to confirm that the project did not meet the criteria for research.

## Results

### Initial steps of the intervention

**Figure 2:**
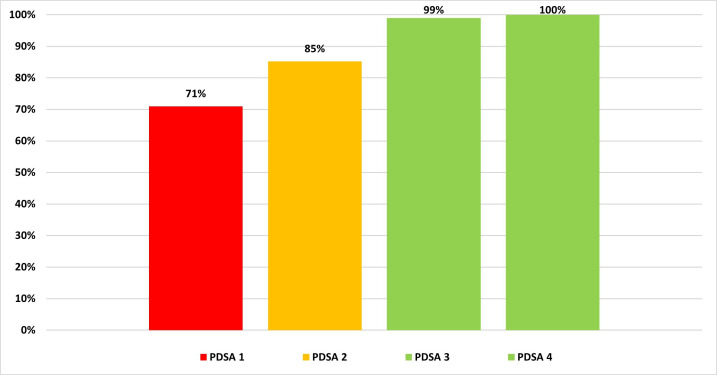
Compliance rates for each Plan–Do–Study–Act (PDSA) cycle in 2024.

**Figure 3:**
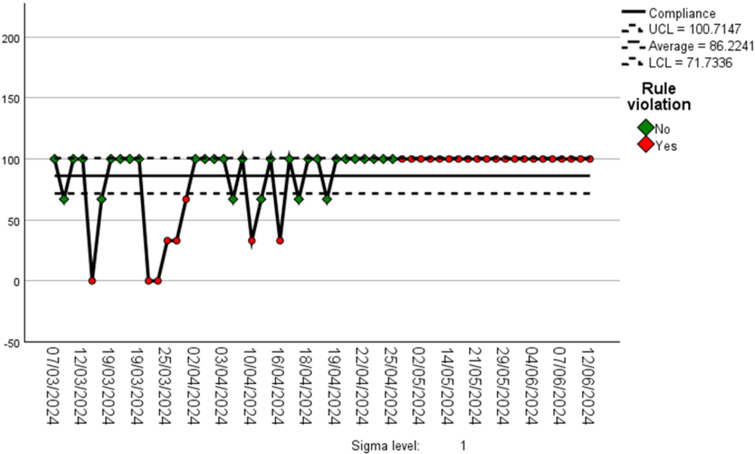
Compliance rates for the quality improvement project. UCL, upper control limit; LCL, lower control limit.

The data are presented using a bar graph (see figure 2) and control chart (see figure 3) of clinician compliance rates to determine change during each cycle. Each cycle was 3 weeks in duration, apart from the PDSA 4 which was extended to 5 weeks, in order to assess sustainability of the results.

PDSA 1 resulted in a small improvement in compliance rates, with compliance increasing from 70% to 71%. However, feedback from clinicians suggested that more practical tools were needed to help apply the guidelines in practice. In response, PDSA 2 introduced the digital folder and clinical flowchart (see figure 1), which provided a more structured approach to decision-making. This intervention led to a significant improvement in compliance rates, with compliance increasing by 14%, from 71% to 85%. This was further improved with the introduction of the quiz in PDSA 3 to 99%, and 100% in PDSA 4.

SPSS^25^ Version 29 was used to plot the statistical processing chart as a control chart, which included the eight Nelson rules.^23^ The QIP control chart highlighted common and special cause variations (see figure 3), and this was analysed. Rules 1 (1 point or more and 3 SDs or more from the mean leading to an outlier), 5 (2 or 3 points or more in a row and more than 2 SDs from the mean in the same direction leading to a shift) and 2 (9 or more points in a row on the same side of the mean causing a shift) contributed to four data outliers and two shift special cause variations (see figure 3).

### Outcome and process measures

The outcome measure demonstrated a 16% improvement in average compliance rates for orthopaedic referrals for the four wrist and hand conditions against EBI and local guidance when comparing the audit data (n=34) with the QIP data (n=58). This figure was 1% higher than the proposed QIP target of 85%.

The first process measure of clinician confidence, as measured by pre-QIP and post-QIP questionnaires, improved by 15%. The second process measure of quiz scores also improved, with the average score increasing by 12% between PDSA 3 and PDSA 4. This improvement in both confidence and knowledge retention suggests that the educational interventions were successful in engaging clinicians and helping them apply the guidelines in practice.

The first balance measure demonstrated a 3.9% increase in the percentage of referrals to orthopaedics for the four wrist and hand conditions. The second balance measure showed a 3.2% increase in referrals to injection clinics, indicating that clinicians were becoming more confident in managing conditions like CTS and trigger finger conservatively. This reduction in surgical referrals for these conditions suggests that the project had a positive impact on reducing unnecessary referrals to secondary care.

### Contextual element interactions

The introduction of the clinical flowchart in PDSA 2 was identified as a key driver of change as it provided clinicians with a clear, easy-to-use tool for making referral decisions. The use of case discussions and quizzes also contributed to the steady improvement in compliance rates as they allowed clinicians to apply their knowledge in real time and receive feedback on their decision-making processes.

By the end of PDSA 4, which was extended to a 5-week period, compliance rates had stabilised at 100%, indicating that improvements were likely to be sustainable in the long term. Further analysis has demonstrated sustained improvement post-QIP. Clinicians reported feeling more confident in applying the guidelines and were more likely to refer patients appropriately, reducing the risk of unnecessary surgeries and improving patient outcomes.

### Observed associations

The increase in compliance rates was accompanied by an increase in the overall volume of referrals, suggesting that improving the compliance of referrals did not result in a reduction in the number of cases being referred. In fact, the increase in referrals to injection clinics suggests that clinicians were more confident in managing cases conservatively, reducing the need for surgical intervention.

The project also demonstrated the value of interactive, small group teaching, as it allowed clinicians to engage with the material in a more meaningful way. The quizzes provided a measurable way to track knowledge retention and helped identify areas where further clarification or additional education was needed.

### Unexpected findings

While the majority of the cases reviewed during the project were for CTS, the improvements in compliance were observed across all four wrist and hand conditions. This suggests that the educational interventions were broadly effective, even for conditions like ganglions and trigger finger, which were encountered less frequently by clinicians.

Some clinicians expressed concerns that the additional guidance and the flowchart might discourage them from making referrals, as they expressed that the process was complex. However, the increase in compliance rates suggests that these concerns were largely unfounded.

### Details of missing data

A remote clinician did not assess as many wrist and hand cases as their colleagues, resulting in missing data for that individual. This did not significantly impact the overall outcomes of the project, as the remaining data were sufficient to draw meaningful conclusions.

## Discussion

### Summary

There was a 30% improvement in compliance rates for wrist and hand referrals to orthopaedics, with a particular strength of the project achieving 100% compliance by the end of the project. The outcome measure demonstrated a 16% improvement from the audit. This was 1% higher than the proposed QIP target of 85%, and these improvements were observed across all four conditions. The improvements were achieved through targeted educational interventions, including case discussions, quizzes and the introduction of a clinical flowchart. The project also improved clinician confidence and knowledge retention, as evidenced by the increase in quiz scores and questionnaire responses.

### Interpretation

The success of the project can be attributed to the structured educational interventions, which effectively engaged clinicians and improved their ability to apply referral guidelines in practice. The combination of small group teaching, real-time case discussions and quizzes provided clinicians with multiple opportunities to reinforce their learning. The introduction of the clinical flowchart in PDSA 2 was particularly effective, as it gave clinicians a practical tool to use during consultations, reducing the risk of inappropriate or incomplete referrals, which may prove to be cost-effective in the long term. The lasting improvements indicated that the changes were likely to be sustainable in the long term, and this sustainability was evidenced post-QIP.

Research specific to improving the compliance of wrist and hand orthopaedic referrals is limited, with much of the existing literature focusing on general orthopaedic referrals or secondary care pathways.

### Limitations

One limitation of the project was that it did not track cases that were not referred, but who should have been referred to orthopaedics, which could have provided additional insights such as if the condition worsened in these cases. Additionally, the timeframes of the audit and the QIP differed, which may have affected the comparability of the data. The project also did not measure diagnostic workup waiting times before referral, or surgery conversion rates, or discharges at the first orthopaedic appointment, or waiting times after referral, which could provide further insights into the quality of referrals.

Although the QIP was centred around guidance, a SDM approach^26^ involving person-centred practice^11^ should be at the heart of any consultation. The aim should be to address the patient’s ideas, concerns and expectations,^27^ and this might not always be possible when following guidance. Especially for patients who do not have capacity or the ability to adhere to conservative measures. None of these challenges were encountered during the data collection of this project. Since submission, the EBI programme has been revised and updated, and the main change is in Dupuytren’s contractures, where the guidance has removed the statement on ‘degrees’, and replaced this with ‘function’. This has also been adopted in local guidance. As a result, this change will help prevent discrimination and ensure that patients are not inappropriately denied referral for this condition.

Future projects could address these limitations by incorporating data on non-referrals and tracking surgery conversion rates. Additionally, conducting a follow-up audit 12 months after the completion of the QIP would help assess the long-term sustainability of the improvements, however improvements were sustained 6 months after completion of the QIP.

### Conclusions

It is well established that adherence to clinical guidance can improve referral compliance for elective surgery.^28^ The QIP successfully improved compliance with national and local referral guidelines for wrist and hand conditions, demonstrating the effectiveness of educational interventions in improving clinical practice. The use of case discussions, quizzes and clinical decision-making tools like flowcharts provides a simple yet effective model that could be replicated in other community MSK services.

More research and QIPs are required; however, the simplicity of the change interventions employed should be encouraging for the wider MSK community settings. As with most healthcare settings, one of the many challenges is time; therefore, simple change interventions are a desirable choice. Further research is needed to explore the long-term sustainability of these improvements and their impact on patient outcomes. Nonetheless, the success of the QIP highlights the importance of targeted, evidence-based interventions in improving healthcare quality and ensuring that patients receive the most appropriate care in a timely manner. The Standards for Quality Improvement Reporting Excellence in Education was a useful tool to use.^29^

## Supplementary material

10.1136/bmjoq-2025-003323 online supplemental file 1

## Data Availability

Data may be obtained from a third party and are not publicly available.
