# Supplementary figures and images for: Improving the compliance of orthopaedic wrist and hand referrals against the musculoskeletal recommendations from the 2018 Evidence-based Interventions programme, along with local guidance in Greater Manchester: A quality improvement project
Source: BMJ Open Qual. 2025 Sep 26;14(3):e003323. doi: 10.1136/bmjoq-2025-003323 (PMC12481375; doi:10.1136/bmjoq-2025-003323)

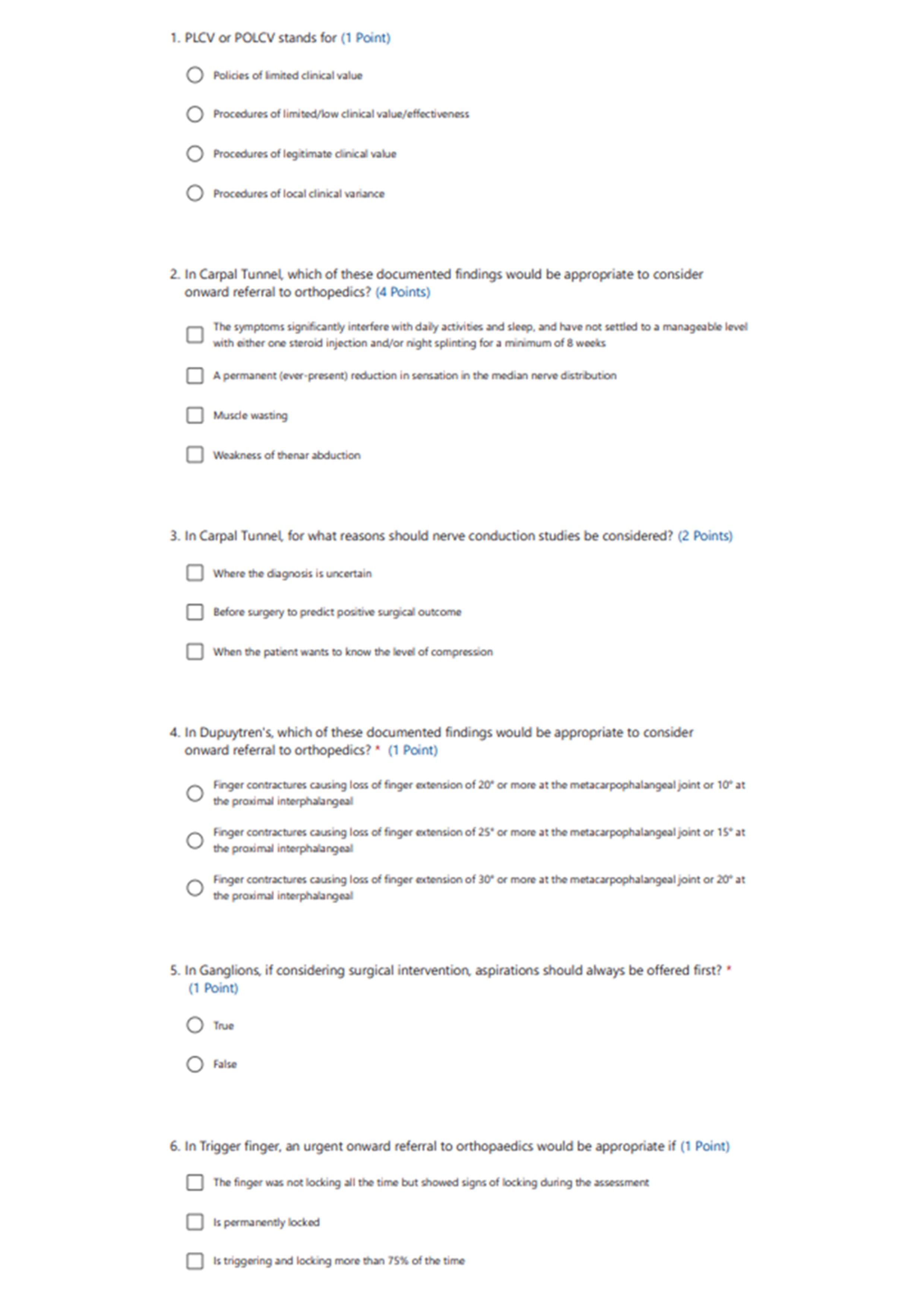

Supplement: online supplemental file 1 [file bmjoq-14-3-s001.jpg]
